# Purulent Pericarditis Identified with Point-of-Care Echocardiography: A Case Report

**DOI:** 10.5811/cpcem.62984

**Published:** 2026-08-05

**Authors:** Daniel Baquet, Aaron Blevins, Dillon Casey, Casey Glass, Jordan Seaback, Jacob Schoeneck

**Affiliations:** * Northeast Georgia Medical Center, Gainesville, Georgia; † Wake Forest University School of Medicine, Department of Emergency Medicine, Winston-Salem, North Carolina

**Keywords:** point-of-care-ultrasound, echocardiography, pericarditis, cardiac tamponade, case report

## Abstract

**Introduction:**

Purulent pericarditis is a rare subset of pericarditis that can result in serious morbidity and mortality. Presenting symptoms are often nonspecific, and a high index of suspicion must be maintained to reach an early diagnosis. Point-of-care ultrasound can be pivotal in the emergency department evaluation of purulent pericarditis, providing key information including characteristics of the effusion and a sonographic assessment for signs of tamponade. We present a case of purulent pericarditis first detected using point-of-care ultrasound in the emergency department.

**Case Report:**

A 55-year-old male presented with chest pain and dyspnea and was found to have cardiac tamponade secondary to purulent pericarditis.

**Conclusion:**

Cases of purulent pericarditis are rare but should be recognized by emergency clinicians. Point-of-care ultrasound is a powerful tool to assist in early diagnosis of pericardial effusion, allowing assessment for signs of cardiac tamponade. Early management with broad spectrum antibiotics and cardiology consultation are mainstays of treatment and can significantly improve outcomes.

## INTRODUCTION

Pericarditis, a condition characterized by inflammation of the pericardium—the double-layered sac surrounding the heart—is often encountered in the emergency department (ED). This inflammatory process has many potential etiologies including infection, autoimmune disease, traumatic injury, or underlying systemic diseases. One rare and intriguing subset of this condition is purulent pericarditis, which presents additional diagnostic and therapeutic challenges. Also known as suppurative pericarditis, purulent pericarditis is notable for its unique precipitants, clinical presentation, and management considerations. Purulent pericarditis is characterized by the presence of pus within the pericardial space, almost exclusively from an infectious source. Although rare, this subset of pericarditis is of distinct clinical significance due to its potential for rapid progression, significant morbidity, and high mortality. Unlike nonpurulent forms, purulent pericarditis often requires urgent and aggressive management because the accumulation of purulent material can lead to cardiac tamponade, circulatory collapse, and other life-threatening complications including septic shock.^1^

The presentation of purulent pericarditis is similar to other forms of pericarditis with initial presenting symptoms including chest pain, fever, and dyspnea. The presence of systemic signs of infection, including sustained high fevers, leukocytosis, or sepsis should increase the suspicion for purulent pericarditis. Confirming the diagnosis can be challenging; it involves a combination of clinical assessment, blood cultures, echocardiography, computed tomography, and pericardial fluid analysis.^2^ Timely diagnosis is crucial to initiate appropriate management, which requires a multidisciplinary approach with rapid consultant involvement. Broad-spectrum antimicrobial agents, depending on the identified pathogen, are the cornerstone of treatment. Drainage of the pericardial effusion via pericardiocentesis or surgical intervention is vital to stabilize hemodynamics and obtain source control.^3^

## CASE REPORT

A 55-year-old male presented to the ED with two days of chest pain, shortness of breath, nausea, intermittent vomiting, and diarrhea. He had a previous medical history of insulin-dependent diabetes and a liver abscess requiring surgical drainage. He had returned to the United States from Mexico two weeks prior and had been noncompliant with his insulin regimen since that time. On arrival to the ED his initial blood pressure was 112/83 mm Hg; heart rate, 138 beats per minute; oral temperature, 98.2 °F; respiratory rate, 20 breaths per minute; and oxygen saturation, 98% on room air. He was pale, ill appearing, and in mild distress, but cooperative and conversant. He was alert and oriented to person, place, and time and his Glasgow Coma Scale (GCS) was 15. Physical examination was notable for clear bilateral breath sounds, no adventitious heart sounds, and mild generalized abdominal tenderness to palpation.

*CPC-EM Capsule*

What do we already know about this clinical entity?

*Purulent pericarditis is a rare but serious subset of pericarditis with high morbidity and mortality. Symptoms are nonspecific.*

What makes this presentation of disease reportable?

*Point-of-care ultrasound (POCUS) was used to rapidly evaluate and diagnose pericardial effusion with early tamponade physiology leading to timely intervention.*

What is the major learning point?

*Point-of-care ultrasound is pivotal in assessing unexplained tachycardia or hypotension and should be used early in the evaluation of patients in the emergency department.*

How might this improve emergency medicine practice?

*The early use of POCUS can be essential in the prompt diagnosis and management of life-threatening conditions.*

The initial team caring for the patient was concerned for diabetic ketoacidosis or hyperglycemic hyperosmolar syndrome, with concurrent underlying infection, severe electrolyte derangements, or acute renal injury. The patient was resuscitated with an intravenous bolus of one-liter normal saline followed by a second liter of lactated Ringer’s. An electrocardiogram (ECG) showed sinus tachycardia with QRS complexes < 10 mm in the precordial leads consistent with low voltages. The ECG did not demonstrate ischemic ST-segment changes or electrical alternans. Initial laboratory testing was notable for the following: hyperglycemia, 718 mg/dL (reference range, 70–99 mg/dL); moderate acidosis, pH 7.23 (7.35–7.45); acute kidney injury, creatinine 4.1 mg/dL (0.60–1.20 mg/dL); elevated blood urea nitrogen, 96 mg/dL (7–25 mg/dL); nonhypotonic hyponatremia due to endogenous glucose with sodium 123 mmol/L (136–145 mmol/L); mild hyperkalemia, potassium 5.5 mmol/L (3.5–5.1 mmol/L); hypochloremia, 90 mmol/L (98–107 mmol/L); diminished bicarbonate, 19 mmol/L (21–31 mmol/L); severe neutrophilic leukocytosis with white blood cell count of 32.9 × 10^3^ cells/μL (4.4–11.0 × 10^3^ cells/μL), and serum lactic acid, 2.1 mmol/L (0.5–2.2 mmol/L). Initial chest radiograph demonstrated mild cardiomegaly and bibasilar atelectasis. On reassessment following initial fluid resuscitation, the patient reported feeling improved with a downtrend in his hyperglycemia from 718 to 555 mg/dL. He was ultimately placed on a fixed-rate insulin infusion at four units per hour given his likely concomitant diabetic ketoacidosis.

**Image 1:**
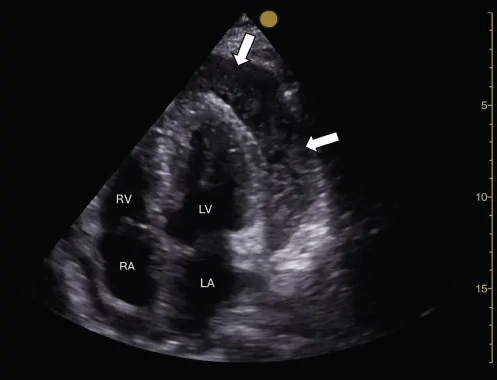
Point-of-care ultrasound showing an apical four chamber view of the patient’s heart with visualization of the left atrium (LA), left ventricle (LV), right atrium (RA), and right ventricle (RV). Note the large mixed-echogenicity pericardial effusion (white arrows).

A point-of-care echocardiogram, performed to evaluate persistent tachycardia and guide fluid resuscitation, showed a large mixed-echogenicity pericardial effusion measuring > 2 cm at the largest point in diastole (Image 1). We also noted mild systolic collapse of the right atrium suggesting sonographic signs of early tamponade. Consultation was made to cardiology with concern for possible cardiac tamponade given the patient’s persistent tachycardia and the findings on point-of-care ultrasound (POCUS). Cardiology reviewed the ultrasound images remotely and agreed with the concern for possible purulence due to the heterogenous echogenicity of the effusion. There was significant concern for intra-abdominal infection given the patient’s history of previous liver abscess with associated abdominal discomfort and gastrointestinal symptoms. Noncontrast computed tomography of the chest, abdomen, and pelvis showed a moderate-to-large pericardial effusion, small bilateral pleural effusions, and small volume abdominopelvic ascites. The patient was admitted to an intermediate care unit.

**Image 2:**
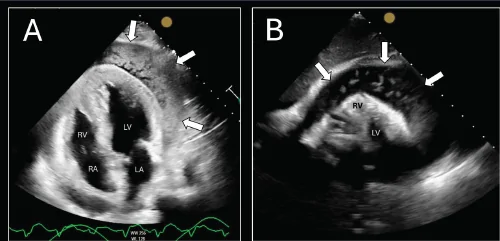
Cardiology ultrasound showing an apical four-chamber view (A) and a subxiphoid view (B) of the patient’s heart with visualization of the left atrium (LA), left ventricle (LV), right atrium (RA), and right ventricle (RV). There is a large mixed-echogenicity pericardial effusion (*white arrows*).

Complete transthoracic echocardiography performed the following day showed a similar mixed-echogenicity pericardial effusion without additional signs of cardiac tamponade (Image 2). Ultrasound clips can be viewed in the supplemental video. Cardiology performed a diagnostic pericardiocentesis for pericardial fluid analysis. were trended, which demonstrated levels of 20, 16, and 20 ng/L, respectively (high sensitivity assay, reference range, < 20 ng/L). Cardiology removed 550 mL of pericardial fluid, and the patient was transferred to a tertiary-care center with the capability to perform a pericardial window procedure. Laboratory results of the pericardial fluid analysis demonstrated positive culture of *Klebsiella variicola*. Species susceptibilities demonstrated the bacteria to be susceptible to amoxicillin-clavulanate, and the antibiotic regimen was managed in consultation with infectious diseases.

## DISCUSSION

Pericarditis is a broad disease category describing inflammation of the pericardium. The pericardial sac is composed of two distinct layers, the serous visceral layer, and the fibrous parietal layer. Between these two layers lies a small layer of physiologic pericardial fluid that functions to provide lubrication and protection for the heart.^4^ Pericarditis is the most common inflammatory disease of the heart with an estimated incidence. of 27.7 cases per 100,000 subjects per year. It is also estimated to be responsible for 5% of ED visits for chest pain.^5^ Pericarditis can be further divided by its acuity (acute, subacute, chronic, or recurrent) and underlying etiology, which can be infectious or non-infectious and occur in isolation or as part of a systemic process. Among infectious etiologies, viral pericarditis is the most common, estimated to account for 80–85% of all cases, vastly outnumbering bacterial, fungal, and parasitic causes.^5^ Non-tuberculosis bacterial pericarditis is rare with an incidence < 1%.^4^,^6^ Other noninfectious etiologies include autoimmune, neoplastic, metabolic (such as uremia), and drug. induced.

Bacterial pericarditis generally occurs as a secondary infection by contiguous spread from a nearby intrathoracic infection with *Streptococci*, *Staphylococci*, *Haemophilus*, and *Mycobacterium tuberculosis* being common isolates. Direct extension from an empyema occurs in approximately 50% of cases and a pneumonia in 33% of cases. Hematogenous dissemination from a distant infection elsewhere in the body can also occur.^3^ Our patient presented with acute purulent pericarditis secondary to underlying *K variicola*. *Klebsiella variicola* is an opportunistic Gram-negative bacteria related to *K pneumonia* and is typically implicated in urinary and respiratory tract infections.^7^ The incidence of *Klebsiella* pericarditis in general is unknown as there is a paucity of literature implicating this organism. Regardless of the organism, treatment of purulent pericarditis should be aggressive with early initiation of broad-spectrum antibiotics and cardiology consultation. Definitive treatment ultimately relies on source control with drainage and possible surgical pericardial window. Intrapericardial thrombolysis is an additional consideration for loculated pericardial effusions.^3^,^4^ Unfortunately, mortality rates approach 40% in treated patients, and death is mostly due to cardiac tamponade or systemic toxicity.^8^

Tamponade physiology occurs when pressures in the pericardial space exceed the pressures in the cardiac chambers resulting in impaired filling pressures during the cardiac cycle. Impairment of cardiac function occurs sequentially with the low-pressure atrial chambers being affected initially and progression to the higher pressure ventricular chambers as the pericardial effusion accumulates. The effect of expanding pericardial fluid is most clearly visualized with ultrasound when the pressure is lowest in the respective chambers, during systole for the atria and diastole for ventricles. The echocardiographic findings concerning for tamponade are summarized in supplemental Appendix A.^9^

In this case, early echocardiographic assessment to evaluate the patient’s persistent tachycardia despite fluid resuscitation allowed for expeditious diagnosis of a pericardial effusion with concern for early tamponade physiology. Rapid cardiac evaluation with POCUS to assess for effusion is an essential tool for all emergency physicians. Additional measures to evaluate for signs of tamponade can allow for early consultation with cardiology and aggressive treatment to avoid cardiovascular collapse. This case highlights the pivotal role of POCUS in the assessment of unexplained tachycardia or hypotension, which allowed for early recognition and management of this patient’s bacterial pericarditis with expeditious antibiotics and pericardiocentesis.

## CONCLUSION

In cases of purulent pericarditis, a vigilant clinical approach coupled with early use of point-of-care cardiac ultrasound is imperative. Swift diagnosis through a heightened index of suspicion facilitates timely intervention and mitigates potential complications. This case underscores the pivotal role of early point-of-care echocardiographic assessment in ensuring prompt and effective management of this life-threatening condition.

## Supplementary Information

Video Transthoracic echocardiography demonstrating a large mixed-echogenicity pericardial effusion with sonographic findings concerning for early cardiac tamponade
