# Supplementary material for: Purulent Pericarditis Identified with Point-of-Care Echocardiography: A Case Report
Source: Clin Pract Cases Emerg Med. 2026 Aug 5;10(3):388–91. doi: 10.5811/cpcem.62984 (PMC13518030; doi:10.5811/cpcem.62984)
Supplement: Supplementary file 1 [file cpcem-10-388-s002.pdf]

Appendix A. Key echocardiographic findings indicative of cardiac tamponade

**Echocardiographic Finding**	**Explanation**
Right atrial systolic collapse for greater than 1/3 of systole	○ Assess in the apical four chamber view<br>○ Can freeze the ultrasound image and assess frame by frame using the cine loop on the machine<br>○ 94% sensitive, 100% specific for tamponade
Right ventricular collapse during diastole	○ Assess in the parasternal long axis or subxiphoid view<br>○ Can use M-Mode tracing over the right ventricle and mitral valve to confirm that ventricular collapse is occurring during diastole (when the mitral valve is open)<br>○ 60% sensitive, 85% specific for tamponade
Reversal of normal septal motion with septal bowing towards the left ventricle during diastole and towards the right ventricle in systole	○ Assess in the apical four chamber view<br>○ Increased right ventricular pressure during inspiration shifts the septum towards the left ventricle during diastole and towards right ventricle during systole (a reversal of normal septal motion)

Decline in left and right ventricular filling with inspiration	○ Assess with a spectral doppler tracing of left or right ventricular inflow<br>○ With inspiration diastolic filling in the right ventricle is augmented but diminished in the left ventricle and flow velocities are augmented in the pulmonary artery but diminished in the aorta<br>○ A greater than 25% decline in peak mitral inflow velocity or a greater than 40% decline in peak tricuspid inflow velocity with inspiration is indicative of cardiac tamponade
Decline in velocity of myocardial movement during early diastole	○ Assess in the apical four chamber view with a tissue doppler tracing over the mitral annulus to measure early filling velocity (e’), which is compared in a ratio to mitral inflow velocity (E) as E/e’<br>○ As myocardial tissue velocity declines the E/e’ ratio increases with values less than 8 considered to be normal and values greater than 15 reflecting an increase in left ventricular filling pressures

Dilation of the Inferior Vena Cava (IVC)	○ Assess maximum IVC diameter and IVC collapsibility index (maximum diameter – minimum diameter/maximum diameter) during inspiration using a longitudinal subcostal view<br>○ An IVC diameter >2.0 cm or collapsibility index less than 20% indicates increased pressure in the IVC<br>○ 97% sensitive, 40% specific for tamponade
